# Determination of a complex crystal structure in the absence of single crystals: analysis of powder X-ray diffraction data, guided by solid-state NMR and periodic DFT calculations, reveals a new 2′-deoxyguanosine structural motif†
†The experimental datasets for this study and the magres output (.magres) files from the CASTEP calculations are available from the Cardiff University data catalogue at http://doi.org/10.17035/d.2017.0031643370

‡
‡Electronic supplementary information (ESI) available. CCDC 1535685. For ESI and crystallographic data in CIF or other electronic format see DOI: 10.1039/c7sc00587c



**DOI:** 10.1039/c7sc00587c

**Published:** 2017-03-16

**Authors:** Colan E. Hughes, G. N. Manjunatha Reddy, Stefano Masiero, Steven P. Brown, P. Andrew Williams, Kenneth D. M. Harris

**Affiliations:** a School of Chemistry , Cardiff University , Park Place , Cardiff , CF10 3AT , UK . Email: HarrisKDM@cardiff.ac.uk; b Department of Physics , University of Warwick , Coventry , CV4 7AL , UK; c Dipartimento di Chimica “G. Ciamician” , Alma Mater Studiorum – Università di Bologna , via San Giacomo , 11-40126 Bologna , Italy

## Abstract

Derivatives of guanine exhibit diverse supramolecular chemistry, with a variety of distinct hydrogen-bonding motifs reported in the solid state, including ribbons and quartets, which resemble the G-quadruplex found in nucleic acids with sequences rich in guanine. Reflecting this diversity, the solid-state structural properties of 3′,5′-bis-*O*-decanoyl-2′-deoxyguanosine, reported in this paper, reveal a hydrogen-bonded guanine ribbon motif that has not been observed previously for 2′-deoxyguanosine derivatives. In this case, structure determination was carried out directly from powder XRD data, representing one of the most challenging organic molecular structures (a 90-atom molecule) that has been solved to date by this technique. While specific challenges were encountered in the structure determination process, a successful outcome was achieved by augmenting the powder XRD analysis with information derived from solid-state NMR data and with dispersion-corrected periodic DFT calculations for structure optimization. The synergy of experimental and computational methodologies demonstrated in the present work is likely to be an essential feature of strategies to further expand the application of powder XRD as a technique for structure determination of organic molecular materials of even greater complexity in the future.

## Abstract 2

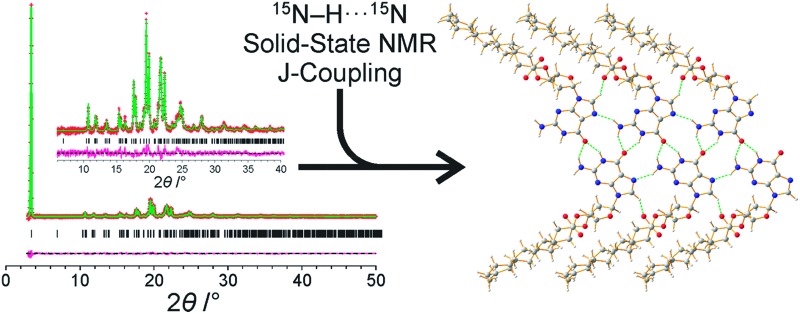
A multi-technique strategy reveals a new hydrogen-bonding motif for a 2′-deoxyguanosine derivative.

## Introduction

Powder X-ray diffraction (XRD) and solid-state NMR spectroscopy are both rich sources of structural data for polycrystalline materials. While successful crystal structure determination of organic molecular solids can now (since the early 1990s) be carried out directly from powder XRD data alone,^1–8^ the structure determination process is often enhanced significantly by consideration of solid-state NMR data for the same material, allowing specific structural details to be established or validated. In general, solid-state NMR data are used in two ways to assist the process of structure determination from powder XRD data.^9^

First, after completing structure refinement (the final stage of structure determination from diffraction data), periodic DFT calculations employing the GIPAW (Gauge Including Projector Augmented Wave) method^10–15^ (for example in the CASTEP program^16^) can be used to calculate solid-state NMR data (*e.g.*, isotropic chemical shifts) for the crystal structure, which may then be compared with the corresponding experimental solid-state NMR data. Clearly, an acceptable level of agreement between calculated and experimental solid-state NMR data can provide strong validation of the crystal structure, augmenting the validation that is already provided by the rigorous assessment^17^ of the quality of fit between experimental and calculated powder XRD patterns in the final Rietveld refinement. This strategy is becoming an increasingly popular way of enhancing the scrutiny and validation of the results obtained in structure determination from powder XRD data.^18–25^

Second, measurements of internuclear couplings from solid-state NMR experiments have the potential to yield information on specific internuclear distances, molecular conformations and/or bonding arrangements in the material. For example, measurement of direct (through-space) dipole–dipole interactions can be used to determine specific internuclear distances in the crystal structure. Measurement of indirect (electron-coupled) dipole–dipole interactions (*i.e.*, *J*-couplings) can also provide useful structural insights that may be utilized in the structure determination process. In this regard, *J*-coupling through hydrogen bonds^26,27^ (*e.g.*, ^15^N···^15^N *J*-coupling in N–H···N hydrogen bonds) can allow the specific functional groups engaged in hydrogen-bonding interactions to be identified. Clearly, such knowledge is particularly valuable in the context of structure determination from powder XRD data, as it may allow plausible structural motifs to be identified in trial structures during the structure solution process or may allow trial structures containing incorrect motifs to be modified or rejected.

**Fig. 1:**
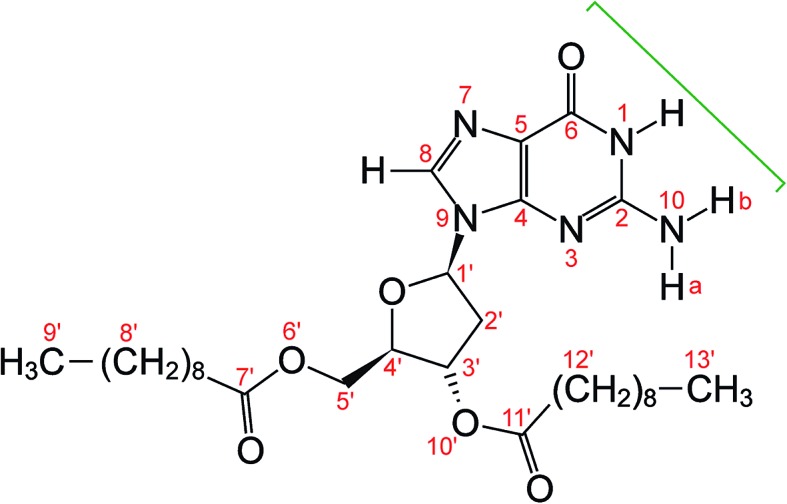
Molecular structure of dG(C_10_)_2_ showing the atom numbering scheme. The green bracket indicates the Watson–Crick hydrogen-bonding groups. The non-hydrogen atoms of the guanine moiety are labelled 1 to 10 and the non-hydrogen atoms of the 2′-deoxyribose moiety are labelled 1′ to 6′ and 10′. Note that the atom labelled here as N10 was labelled N2 or NH_2_ in previous publications^28–30^ on dG(C_10_)_2_.

This paper is focused on structure determination directly from powder XRD data in tandem with consideration of solid-state NMR data, specifically to elucidate the structure of 3′,5′-bis-*O*-decanoyl-2′-deoxyguanosine [denoted dG(C_10_)_2_; Fig. 1]. This material is believed to be polymorphic, as two distinct solid forms have been identified on crystallization from ethanol. In previous work, Pham *et al.* ^28^ referred to these two forms as **2**q and **2**r. The material studied in the present work corresponds to **2**q, as the powder XRD data matches the powder XRD data for **2**q published previously.^29^ We note that **2**q appears to be more readily obtained, as **2**r has only been reported once.^28^ In order to introduce a systematic nomenclature, we define polymorph I of dG(C_10_)_2_ as **2**q and we define polymorph II of dG(C_10_)_2_ as **2**r.

The dG(C_10_)_2_ molecule has found applications in the context of photoelectric devices, including photoconductive materials,^31–33^ biphotonic quantum dots^34^ and photodetectors with rectifying properties.^35^ It has also been shown^36^ that dG(C_10_)_2_ can reversibly interconvert between quartets and ribbons, using a cryptand for cation capture and addition of acid to release the cation. In all these applications, the hydrogen bonding of the guanine moieties is a key factor, emphasizing the importance of understanding the preferred structural properties of dG(C_10_)_2_ in the solid state. Among 3′,5′-bis-*O*-alkanoyl derivatives of 2′-deoxyguanosine, crystal structures have been reported previously only for 3′,5′-bis-*O*-acetyl-2′-deoxyguanosine [dG(C_2_)_2_]^37^ and 3′,5′-bis-*O*-propanoyl-2′-deoxyguanosine [dG(C_3_)_2_],^38^ although several 3′,5′-bis-*O*-silyl derivatives have also been studied^39,40^ and self-assembly of 2′-deoxyguanosine derivatives in solution has been investigated.^41–44^

**Fig. 2:**
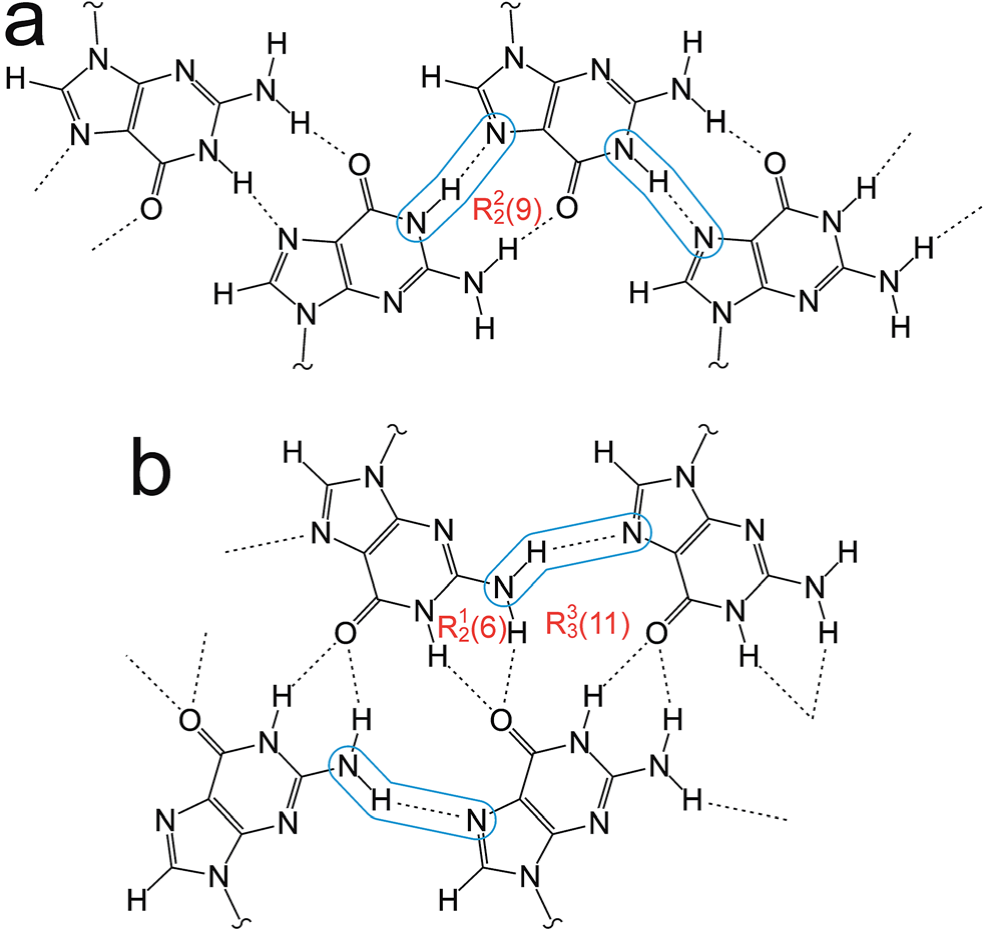
(a) The “narrow” guanine ribbon and (b) the “wide” guanine ribbon. In each case, the N–H···N hydrogen bonds are highlighted and the graph sets^45,46^ for the hydrogen-bonded rings are indicated.

Guanine derivatives are known for their rich supramolecular chemistry.^47–49^ In the solid state, a variety of distinct hydrogen-bonding motifs have been reported, including ribbons and quartets, which resemble the G-quadruplex^50^ found in nucleic acids with sequences rich in guanine. Most reported ribbon motifs are the so-called “narrow” form (Fig. 2a), in which neighbouring guanine moieties are linked by two hydrogen bonds (N–H···N and N–H···O), with each pair of guanines forming a hydrogen-bonded ring designated as *R*22(9) in graph-set notation.^45,46^ A less common motif, described as a “wide” ribbon (Fig. 2b), has been observed in two structures [2′,3′-*O*-bis(tri-isopropylsilyl)guanosine^40^ and 9-(2,3-bis(hydroxymethyl)cyclobutyl)-guanine^51^] and contains three distinct hydrogen bonds: two N–H···O hydrogen bonds between the O atom of one guanine moiety and two different N atoms of a neighbouring guanine moiety [forming a ring with graph set *R*12(6)], and an N–H···N hydrogen bond which, together with the two N–H···O hydrogen bonds, forms a ring involving three guanine moieties with graph set *R*33(11). Another ribbon motif has been observed in the solution state^41,43^ and in a number of salts of 7-methylguanine^52^ in which two distinct hydrogen-bonded rings alternate along the ribbon. Among the reported quartet motifs, there are only two cases^53,54^ in which the quartet is not formed around a metal cation.

As dG(C_10_)_2_ was obtained in our work only as a fine powder (by crystallization from ethanol), powder XRD provides the only viable approach for structure determination. As demonstrated over the past 20 years or so,^1–8^ crystal structure determination of organic materials directly from powder XRD data has become a relatively mature field. Nevertheless, challenges in structure determination can be encountered in specific cases, which can be greatly facilitated by incorporating other sources of information (*i.e.*, other experimental data and/or computational insights) within the structure determination process. As illustrated by the present study of structure determination of dG(C_10_)_2_ – a molecule with 90 atoms – the successful application of techniques for structure determination from powder XRD data is not just limited to the case of relatively small molecules.

## Methods

The sample of dG(C_10_)_2_ was prepared using the method described previously^28^ and crystallized from ethanol. Powder XRD data confirmed that the sample was polymorph I, as defined above. High-quality powder XRD data suitable for structure determination were recorded (at 21 °C), for a powder sample contained in two flame-sealed capillaries, using a Bruker D8 Diffractometer (Ge-monochromated CuKα_1_ radiation) operating in transmission mode with a Våntec detector covering 3° in 2*θ*. The data were recorded in the 2*θ* range from 3° to 50° (step size 0.017°) with a total data collection time of 57 h. A two-dimensional powder XRD pattern was also recorded at ambient temperature using an Agilent SuperNova Dual Atlas diffractometer (CuKα radiation, *λ* = 1.54180 Å) in order to assess the extent of “preferred orientation” in the powder sample.^55^

Periodic DFT calculations for geometry optimization and calculation of NMR parameters were carried out using the CASTEP program^16^ (Academic Release version 8.0). Geometry optimization used ultrasoft pseudopotentials,^56^ PBE functional,^57^ semiempirical dispersion corrections (TS correction scheme^58^), fixed unit cell, preserved space group symmetry and periodic boundary conditions. Isotropic NMR chemical shifts were calculated using the GIPAW approach,^10–14^ while *J*-coupling values were calculated at the scalar-relativistic level of theory using the ZORA method.^59–61^ All calculations used a basis set cut-off energy of 700 eV and a Monkhorst–Pack grid^62^ of minimum sample spacing 0.05 × 2π Å^–1^. In the first instance, chemical shifts are referenced using the formula

1 *δ*_iso_(calc) = *σ*_ref_ – *σ*_iso_(calc)

where *σ*_ref_ is the sum of the mean of the calculated shielding values and the mean of the experimental chemical shifts.^11^ A second referencing method uses the formula

2 *δ*_iso_(calc) = *σ*_0_ – *m σ*_iso_(calc)

with the values of *σ*_0_ and *m* obtained from a least-squares fitting procedure to optimize the agreement between calculated and experimental chemical shifts.

## Structure determination

**Fig. 3:**
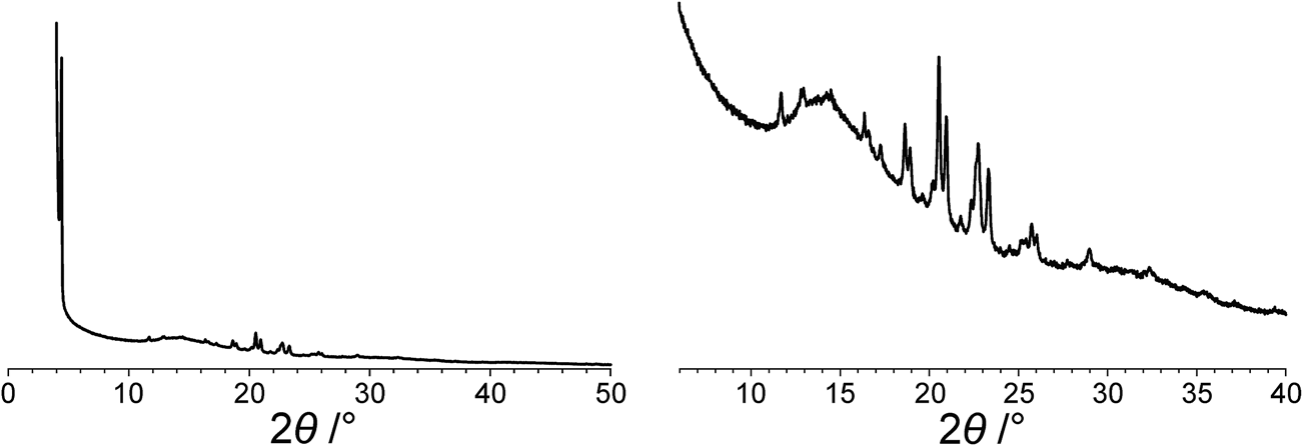
The experimental powder XRD pattern for polymorph I of dG(C_10_)_2_. The full powder XRD pattern is shown on the left; the expanded region from 2*θ* = 6° to 40° is shown on the right.

The powder XRD pattern of polymorph I of dG(C_10_)_2_ was indexed using the DICVOL91 algorithm^63^ in the program Crysfire,^64^ giving the following unit cell with monoclinic metric symmetry: *a* = 8.33 Å, *b* = 7.82 Å, *c* = 25.79 Å, *β* = 97.5°. However, in the subsequent profile-fitting stage of the structure determination process, progress was hampered significantly by two specific features of the powder XRD pattern of dG(C_10_)_2_, which introduced challenges in achieving an acceptable quality of profile fitting (Fig. 3). First, the powder XRD pattern of dG(C_10_)_2_ contains a very intense peak at low diffraction angle (2*θ* = 3.4°), which is substantially more intense than any of the other peaks. The presence of one peak of dominant intensity initially raised the possibility that the powder XRD data may be strongly affected by preferred orientation of the crystallites in the sample. However, the powder XRD pattern (see Fig. S1 in ESI‡) recorded using a two-dimensional detector (for one of the two capillaries used to record the one-dimensional powder XRD data in Fig. 3) exhibited uniform intensity around the Debye–Scherrer rings, indicating that the distribution of crystallite orientations in the powder sample was essentially random and hence that there was no significant preferred orientation.

The second challenging aspect concerns the very high background in the low-angle region of the powder XRD pattern, arising from a significant amount of X-ray scattering from air in the region of the peak at 2*θ* = 3.4°. To achieve a high quality of fit in the profile-fitting stage, which was carried out using the Le Bail technique^65^ in the GSAS program,^66^ it was necessary first to fit the baseline of the low-angle region (2*θ* = 3° to 5°) using a polynomial, which was then subtracted from the experimental data. Although this procedure introduced some artefacts to the baseline, these artefacts were fitted successfully by the shifted Chebyshev polynomials^67^ used for baseline correction in the Le Bail fitting procedure in GSAS. We note that attempts to fit the original baseline using this method were not successful.

**Fig. 4:**
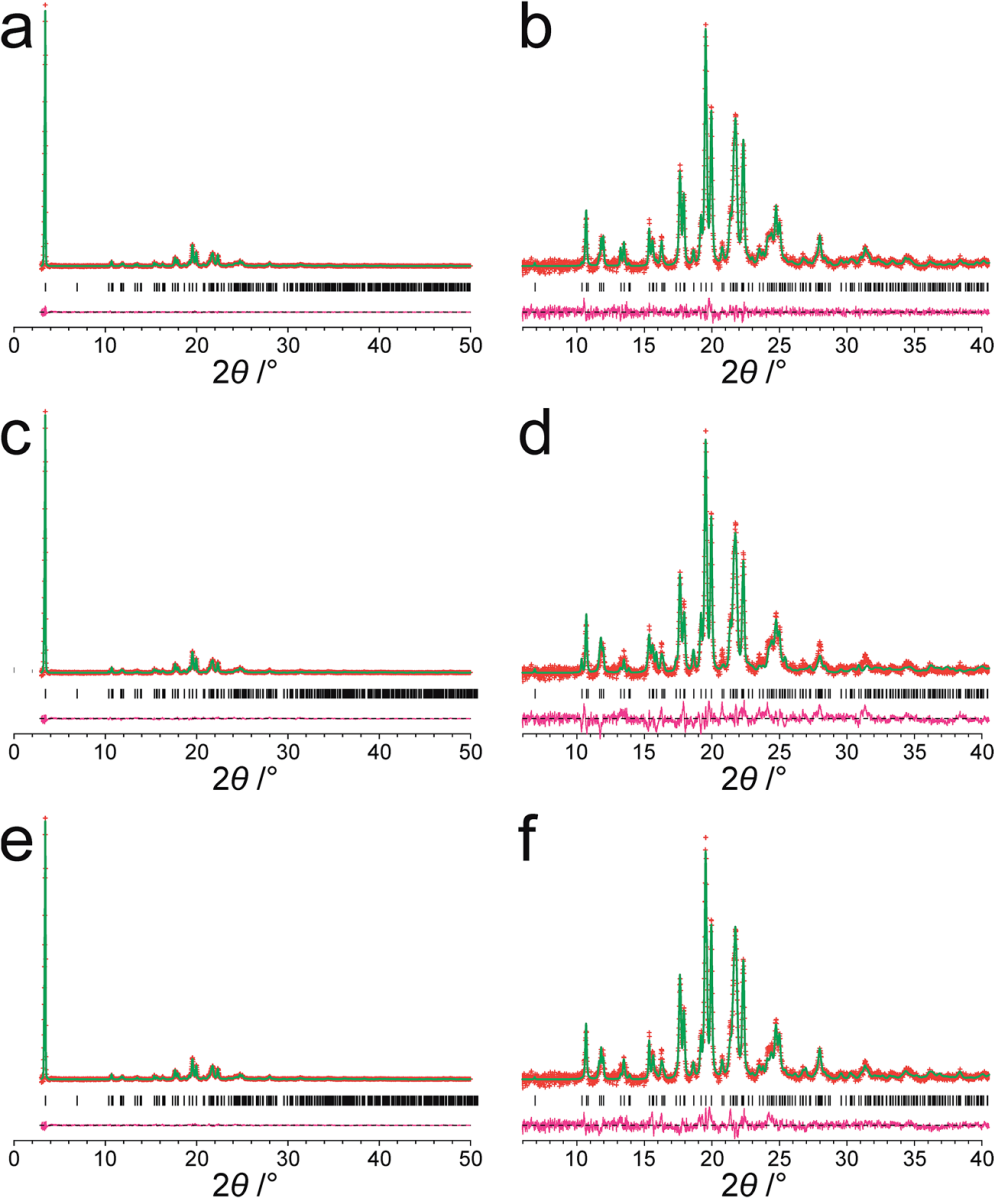
Results from fitting the powder XRD pattern (with baseline subtracted) for polymorph I of dG(C_10_)_2_. (a and b) Results from profile fitting using the Le Bail technique. (c and d) Results from the initial Rietveld refinement discussed in the text. (e and f) Results from the final Rietveld refinement following structure optimization using periodic DFT calculations. The full powder XRD pattern is shown in (a), (c) and (e). The expanded region from 2*θ* = 6° to 40° is shown in (b), (d) and (f). Red + marks, experimental data; green line, calculated data; magenta line, difference plot; black tick marks, peak positions.

The modified powder XRD data were then subjected to Le Bail fitting (Fig. 4a; due to the high intensity of the first peak relative to all other peaks, the data between 2*θ* = 6° and 40° are shown separately with an expanded intensity scale in Fig. 4b). The lineshape of the first peak is rather poorly fitted as a consequence of the double baseline fitting described above. Nevertheless, the overall quality of fit obtained in the Le Bail fitting is considered acceptable (Fig. 4a; *R*_p_ = 0.93%, *R*_wp_ = 1.23%).

Density considerations suggest that there are two molecules of dG(C_10_)_2_ in the unit cell and, given the fact that the dG(C_10_)_2_ molecule is chiral, the only plausible space groups are *P*2 and *P*2_1_. As these space groups could not be distinguished definitively on the basis of systematic absences in the powder XRD data [although the absence of the (010) peak may point towards *P*2_1_], each of these space groups was considered in independent structure-solution calculations using the direct-space genetic algorithm technique^68–70^ in the program EAGER.^71–76^ Structure-solution calculations for space group *P*2 did not generate any plausible trial structures and only space group *P*2_1_ was considered further.

Previous solid-state NMR studies of dG(C_10_)_2_ provide direct structural insights concerning the hydrogen-bonding between guanine moieties. Pham *et al.* ^28,30^ determined the ^15^N chemical shifts and *J*-couplings for polymorph I of dG(C_10_)_2_ (see Table 1), including a ^2h^ *J*_N7N10_ coupling of 5.9 Hz, while Webber *et al.* ^29^ reported ^1^H and ^13^C chemical shifts and found evidence for several H···H short contacts. The value of ^2h^ *J*_N7N10_ provides a strong indication that there is a relatively strong N–H···N hydrogen bond involving N7 and N10, which provided a robust criterion for acceptance or rejection of trial structures obtained in the structure solution from powder XRD data reported here (particularly as a basis for rejecting trial structures that clearly do not contain this hydrogen bond, as discussed in more detail below). Furthermore, comparison of the chemical shifts and *J*-couplings calculated for the final refined crystal structure with the chemical shifts and *J*-couplings measured experimentally provides additional scrutiny and validation of the crystal structure following the final Rietveld refinement.

**Table 1:** Calculated and experimental *J*_NN_-couplings for polymorph I of dG(C_10_)_2_

Coupling	*J*_NN_/Hz	
	Calculated	Experimental^*a*^
^3^ *J*_N3N9_	4.20	3.5^*b*^, 3.8^*b*^
^3^ *J*_N3N10_	5.45	5.3
^2h^ *J*_N7N10_	7.10	5.9

^*a*^Taken from Pham *et al.* (2007).^30^

^*b*^The two values correspond to separate measurements on each resonance.

In setting up the structural model to be used in the direct-space genetic algorithm structure solution calculations, the dG(C_10_)_2_ molecule was constructed as follows. The geometry of the guanine moiety was modelled on the structure of one of the molecules in the reported crystal structure^77^ of guanosine dihydrate (CCDC ref. code GUANSH10) and the geometry of the 2′-deoxyribose ring was modelled on that in the reported crystal structure^38^ of dG(C_3_)_2_ (CCDC ref. code MOFBUE). The two C_10_ chains were constructed using the average bond lengths and bond angles for similar moieties determined using the program Mogul version 1.7.1 (for bonds not involving hydrogen) and from Allen *et al.* ^78^ (for bonds involving hydrogen). The conformation of the 2′-deoxyribose ring was kept fixed during the structure solution calculation. As the position along the *b*-axis can be fixed arbitrarily for space group *P*2_1_, each trial structure was defined by a total of 27 structural variables (2 positional, 3 orientational and 22 torsional variables). The 22 torsional variables are specified in Fig. S2.‡

With this model, the structure solution calculations in space group *P*2_1_ generated trial structures that were considered plausible, including a geometric relation between N7 and N10 consistent with N–H···N hydrogen bonding. In contrast, structure solution calculations using other models for the dG(C_10_)_2_ molecule (*e.g.*, with the geometry of the 2′-deoxyribose ring based on the average bond lengths and bond angles for similar moieties) led to trial structures that were considered implausible as they did not contain hydrogen bonding between N7 and N10.

The genetic algorithm structure solution calculations in space group *P*2_1_ involved the evolution of 32 independent populations of 500 structures, with 50 mating operations and 250 mutation operations carried out per generation, and a total of 500 generations in each calculation. In two of the calculations, the trial structure giving the best quality of fit between calculated and experimental powder XRD data was essentially the same structure, and the quality of fit was significantly better than the best-fit structure obtained in any of the other calculations (see ESI‡ for more details). The trial structure giving the best quality of fit from all the structure-solution calculations was used as the initial structural model for Rietveld refinement,^79^ which was carried out using the GSAS program.^66^ In the Rietveld refinement, restraints were applied to bond lengths and bond angles based on the initial molecular model (discussed above) and planar restraints were applied to the guanine moiety and the two carbonyl moieties. These restraints were relaxed over the course of the refinement. A common isotropic atomic displacement parameter was refined for all non-hydrogen atoms and the value for hydrogen atoms was set equal to 1.2 times the refined value for non-hydrogen atoms. No corrections were applied for preferred orientation. The Rietveld refinement at this stage gave a reasonably good fit to the powder XRD data (Fig. 4c; *R*_p_ = 1.35%, *R*_wp_ = 1.86%).

The structure obtained in this Rietveld refinement was then subjected to geometry optimization using the CASTEP program, leading to small shifts in atomic positions with an average atomic displacement of 0.65 Å. The most significant structural changes concerned the orientations of the two carbonyl moieties in the decanoyl chains. The structure obtained following geometry optimization was then used as the starting structural model for a final Rietveld refinement, which gave an improved fit (Fig. 4e; *R*_p_ = 1.15%, *R*_wp_ = 1.56%) compared to the first Rietveld refinement discussed above. The final refined unit cell parameters were: *a* = 8.3072(7) Å, *b* = 7.8052(10) Å, *c* = 25.7246(27) Å, *β* = 97.491(4), *V* = 1653.73(31) Å^3^ (2*θ* range, 3–50°; 2755 profile points; 289 refined variables). Overall, the combination of geometry optimization followed by further Rietveld refinement led to an average atomic displacement of 0.71 Å, with significant changes in the conformations of the decanoyl chains (particularly in the region of the carbonyl moieties) and a small shift of the 2′-deoxyguanosine moiety, which led to an improvement in geometrical aspects of the hydrogen bonding between guanine moieties in neighbouring molecules.

## Discussion

**Fig. 5:**
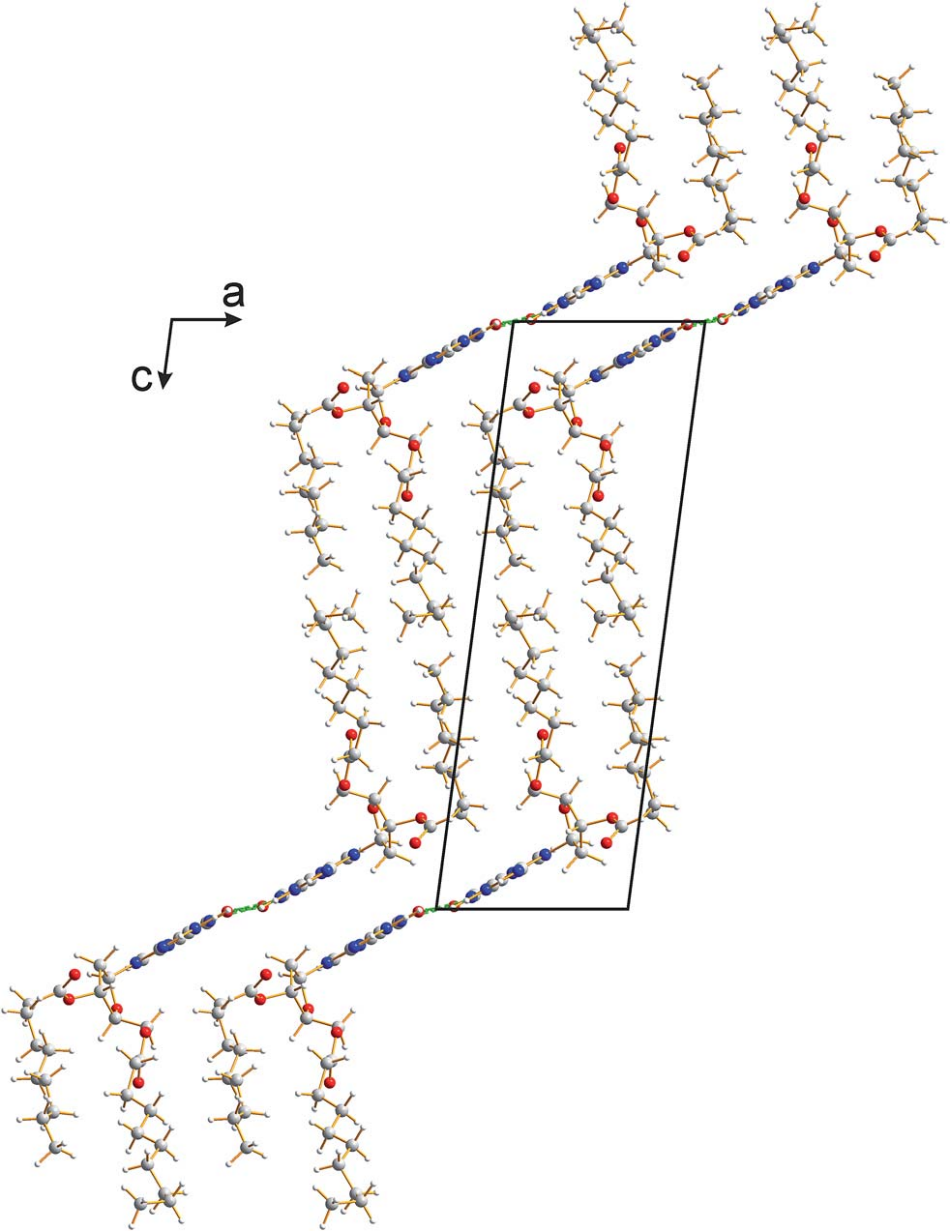
Crystal structure of polymorph I of dG(C_10_)_2_ viewed along the *b*-axis (parallel to the direction of the hydrogen-bonded ribbons).

**Fig. 6:**
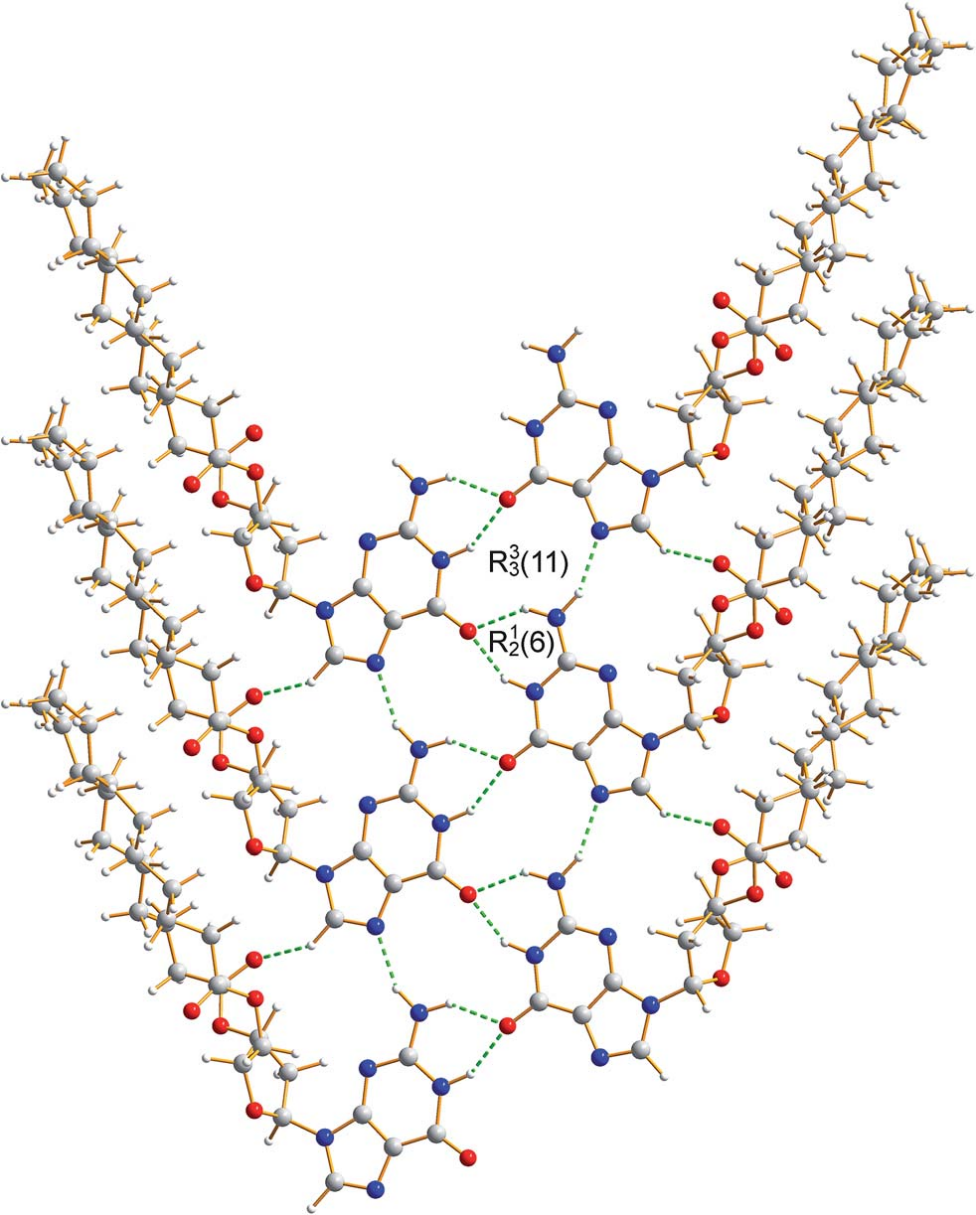
Crystal structure of polymorph I of dG(C_10_)_2_ showing the hydrogen-bonded ribbon of the guanine moieties. In this view, the *b*-axis is vertical.

The crystal structure from the final Rietveld refinement is shown in Fig. 5 and 6. Viewed along the *b*-axis (Fig. 5), it is clear that the structure comprises hydrogen-bonded ribbons constructed from the guanine moieties (the view in Fig. 5 is parallel to the plane of the ribbons). The ribbons run parallel to the *b*-axis and the guanine moieties within a given ribbon are related by the 2_1_ screw axis. The 2′-deoxyribose moiety and alkyl chains occupy the space between adjacent ribbons. The ribbons involve three distinct hydrogen bonds: two N–H···O hydrogen bonds involving N–H10b and N–H1 of a given molecule as donors and the C=O group of a neighbouring guanine moiety as the acceptor, and an N–H···N hydrogen bond involving N–H10a as the donor and the N7 atom of a neighbouring guanine moiety as the acceptor (geometric data for these hydrogen bonds are given in Table S1 in ESI‡). The ribbons in dG(C_10_)_2_ are unambiguously identified (compare Fig. 6 and 2b) as the “wide” ribbon motif,^40,51^ which has not been observed previously for any 2′-deoxyguanosine derivative. A C–H···O hydrogen bond is also identified (see Fig. 6) between C8 of the guanine moiety as the C–H donor and the C=O group containing C11′ of a neighbouring molecule as the acceptor.

**Fig. 7:**
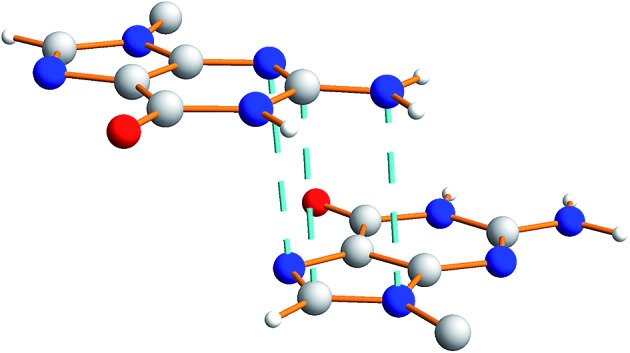
Illustration of π···π interactions between guanine moieties in the crystal structure of polymorph I of dG(C_10_)_2_. The dashed lines represent distances of *ca.* 3.5 Å.

There is also evidence for π···π interactions between guanine moieties in adjacent ribbons in the crystal structure of dG(C_10_)_2_, as the distances from the N3, C2 and N10 atoms of one guanine moiety to the N7, C8 and N9 atoms, respectively, of a neighbouring molecule are all *ca.* 3.5 Å (Fig. 7). Such π···π interactions are not observed in the two previously reported crystal structures^40,51^ containing the “wide” ribbon motif.

The relative arrangement of the 2′-deoxyribose and guanine moieties around the *N*-glycosidic bond (N9–C1′) corresponds to the *syn* conformation, with the Watson–Crick hydrogen-bonding groups (see Fig. 1) directed towards the 2′-deoxyribose ring.^80^ Significantly, Webber *et al.* ^29^ predicted that the crystal structure of dG(C_10_)_2_ should exhibit this structural feature, based on the high values of isotropic ^13^C chemical shift for C8 and C1′, which are characteristic of the *syn* conformation. It is noteworthy that the only guanosine derivative that forms the “wide” ribbon motif in its crystal structure also has the *syn* conformation.^40^

**Fig. 8:**
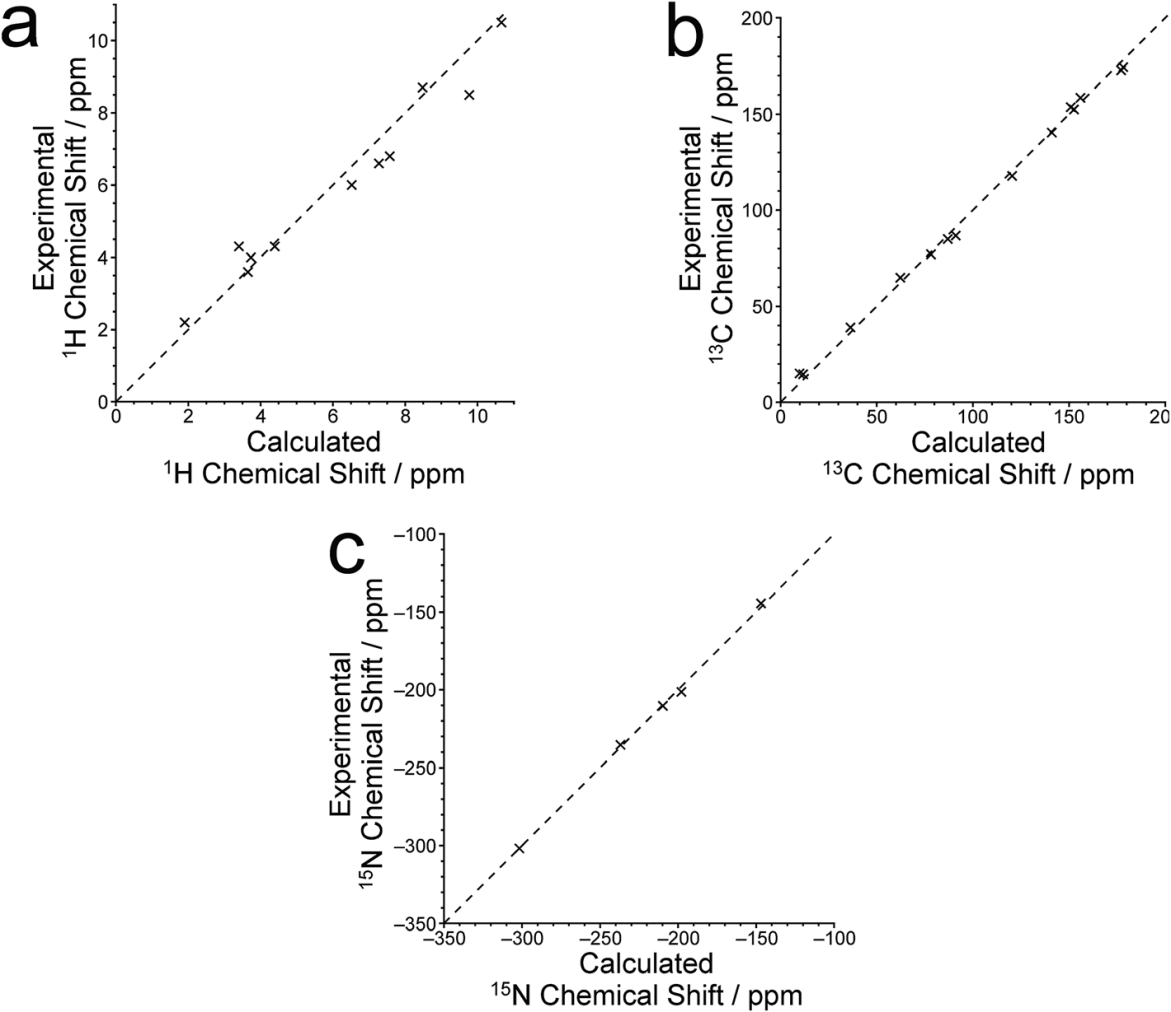
Correlation plots for the calculated and experimental values of the (a) ^1^H, (b) ^13^C and (c) ^15^N isotropic chemical shifts for polymorph I of dG(C_10_)_2_. In each case, the dashed line corresponds to *δ*_iso_(expt) = *δ*_iso_(calc).

The isotropic ^1^H, ^13^C and ^15^N chemical shifts calculated using the CASTEP program for the crystal structure of polymorph I of dG(C_10_)_2_ determined here are compared with the experimental values^29,30^ in Fig. 8 (see also Tables S2–S4‡). The calculated (using eqn (1)) and experimental data are in very good agreement, with RMS deviations of 0.57 ppm, 3.02 ppm and 2.01 ppm for the ^1^H, ^13^C and ^15^N chemical shifts, respectively. From Fig. 8b, it is evident that the calculated ^13^C chemical shifts are higher than the experimental data for the resonances at high ppm and lower than the experimental data for the resonances at low ppm. This phenomenon is well known and can be addressed empirically either by establishing the calculated chemical shifts using eqn (2) and the least-squares fitting procedure (in which the gradient *m* may deviate from unity) described in the Methods section, or by using different reference shieldings for the high-ppm region and the low-ppm region of the spectrum.^11,23,81^ As shown in Fig. S3,‡ when the calculated ^13^C chemical shifts are established using eqn (2), the RMS deviation between calculated and experimental ^13^C chemical shifts is decreased to 2.51 ppm. Using the same procedure (based on eqn (2)) to establish the calculated ^1^H and ^15^N chemical shifts, the RMS deviations between calculated and experimental data are decreased to 0.39 ppm and 1.99 ppm, respectively.

Hartman *et al.* ^82^ have reported that GIPAW calculations of ^13^C chemical shifts across a range of small organic molecules give an RMS deviation of 2.12 ppm when using the procedure based on eqn (2). Although this deviation is slightly lower than that obtained for our results, it is important to note that the dG(C_10_)_2_ molecule is significantly larger and more flexible than any of the molecules considered by Hartman *et al.* Furthermore, the slightly higher RMS deviation observed for dG(C_10_)_2_ may be caused, in part, by the fact that ^13^C chemical shifts for the CH_2_ moieties were not included in our analysis as the ^13^C resonances for individual CH_2_ moieties are not resolved in the experimental ^13^C NMR spectrum.

Three ^15^N···^15^N *J*-couplings across N–H···N hydrogen bonds between guanine moieties were also calculated (see Table 1), specifically the intramolecular couplings ^3^ *J*_N3N10_ and ^3^ *J*_N3N9_, and the intermolecular coupling ^2h^ *J*_N7N10_. In each case, the calculated *J*-coupling is higher, to a greater or lesser extent, than the experimental value, but the calculated values successfully reflect the correct trend.

## Concluding remarks

Several aspects of the structure determination of dG(C_10_)_2_ from powder XRD data reported in this paper presented challenges, including the presence of the very intense (001) peak at low angle in the powder XRD pattern on a very steeply sloping baseline. This peak represents more than 45% of the total diffraction intensity across the 2*θ* range recorded and it was essential to ensure that the method applied for baseline correction did not significantly distort this peak. This factor, combined with the complexity of the direct-space search involved in the structure-solution calculation (a consequence of the large size and flexibility of the dG(C_10_)_2_ molecule), led to a large number of distinct trial structures giving similar fits to the data. In order to identify the correct structure solution, information obtained in previous solid-state NMR studies of dG(C_10_)_2_ proved to be vital, in particular the knowledge that a strong intermolecular N–H···N hydrogen bond exists between N7 and N10. After initial Rietveld refinement, geometry optimization using periodic DFT calculations (with fixed unit cell) generated a structure which, upon further Rietveld refinement, gave an improved fit to the experimental powder XRD data, illustrating the utility of introducing geometry optimization as a key step in the overall structure elucidation process. Clearly, the fact that a wide range of solid-state NMR parameters calculated from the final refined crystal structure are in good agreement with the corresponding experimental solid-state NMR parameters gives additional support to the veracity of the structure determined from powder XRD data. The synergy of experimental and computational methodologies demonstrated in the present work is likely to be an essential feature of strategies to further expand the application of powder XRD as a technique for structure determination of organic molecular materials of even greater complexity in the future.
