# Supplementary material for: Determination of a complex crystal structure in the absence of single crystals: analysis of powder X-ray diffraction data, guided by solid-state NMR and periodic DFT calculations, reveals a new 2′-deoxyguanosine structural motif
Source: Chem Sci. 2017 Mar 16;8(5):3971–9. doi: 10.1039/c7sc00587c (PMC5433513; doi:10.1039/c7sc00587c)
Supplement: Supplementary file 1 [file SC-008-C7SC00587C-s001.pdf]

Electronic Supplementary Material (ESI) for Chemical Science.
This journal is © The Royal Society of Chemistry 2017

Electronic Supplementary Information

Determination of a Complex Crystal Structure in the Absence of Single Crystals: Analysis of Powder X-ray Diffraction Data, Guided by Solid-State NMR and Periodic DFT Calculations, Reveals A New 2'-Deoxyguanosine Structural Motif

Colan E. Hughes,[1] G. N. Manjunatha Reddy,[2] Stefano Masiero,[3] Steven P. Brown,[2] P. Andrew Williams[1] and Kenneth D. M. Harris*[1]

1 School of Chemistry, Cardiff University, Park Place, Cardiff, CF10 3AT, U.K.

2 Department of Physics, University of Warwick, Coventry, CV4 7AL, U.K.

3 Dipartimento di Chimica "G. Ciamician", Alma Mater Studiorum – Università di Bologna, via San Giacomo, 11-40126 Bologna, Italy.

* Author for correspondence: HarrisKDM@cardiff.ac.uk

Additional Figures

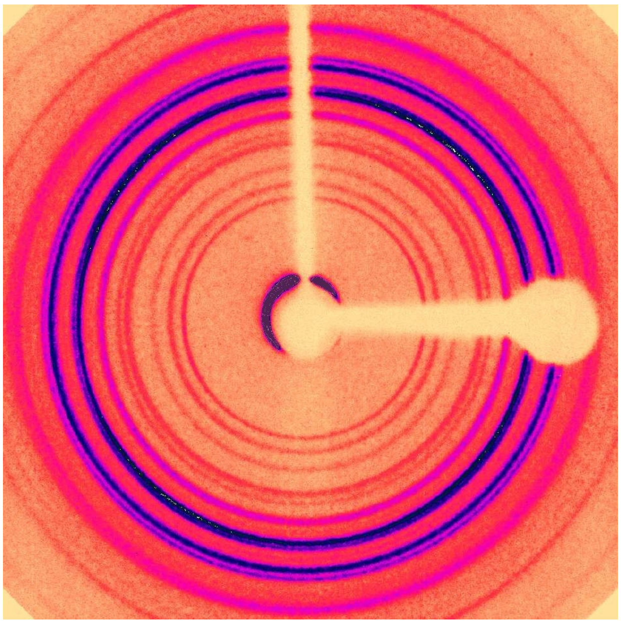

Figure S1. Powder XRD data for polymorph I of dG(C_{10})$_2$ recorded on a two-dimensional detector.

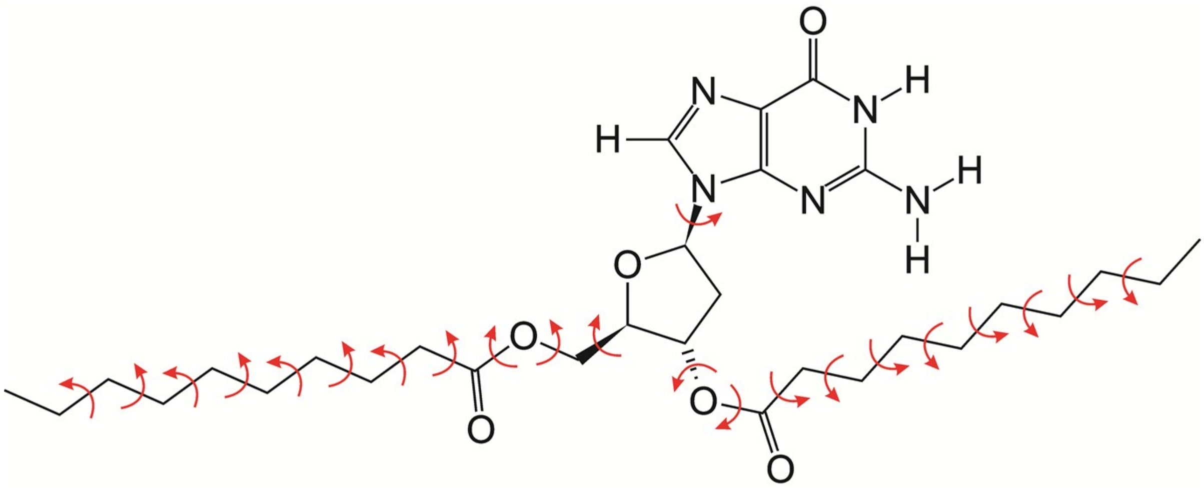

Figure S2. Molecular structure of dG(C_{10})$_2$ showing the 22 torsional variables in the direct-space structure solution calculations.

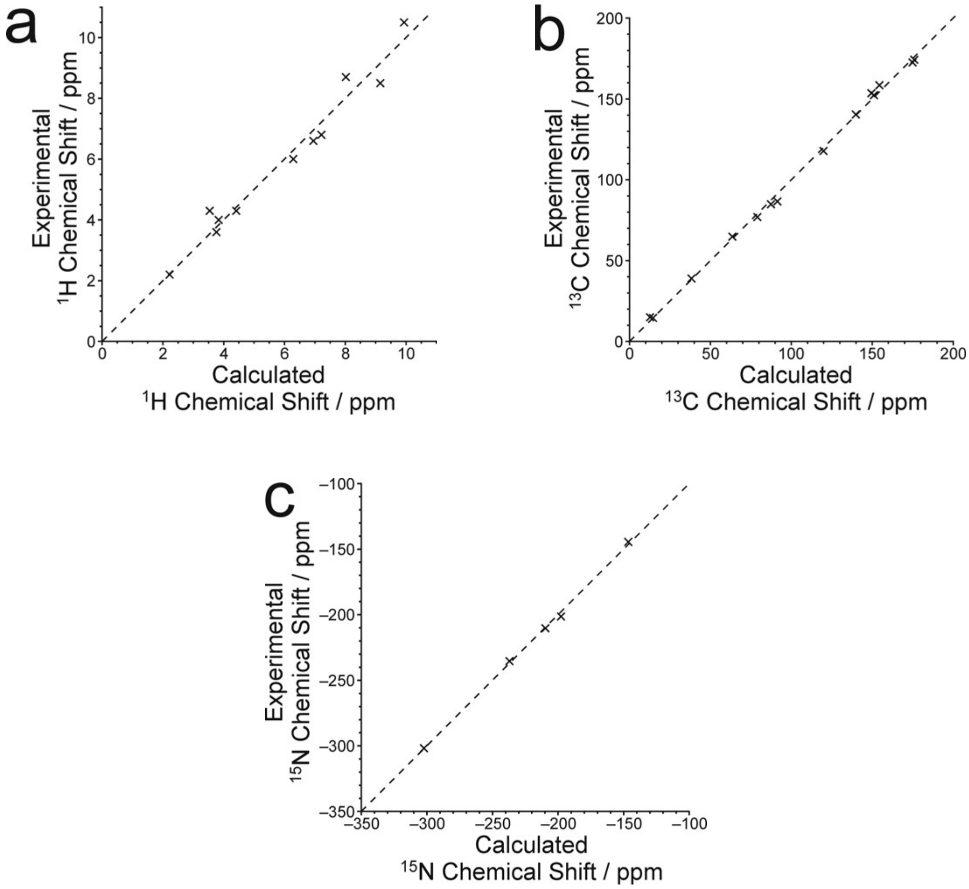

Figure S3. Correlation plots for the calculated and experimental values of the (a) ^{1}H, (b) ^{13}C and (c) ^{15}N isotropic chemical shifts for polymorph I of dG(C_{10})$_2$, in which the calculated chemical shifts were established using Eq. 2 and the least-squares fitting procedure described in the main text. The best-fit values of σ_0 and m (see Eq. 2) obtained were: for ^{1}H, σ_0 = 27.01 ppm and m = 0.881; for ^{13}C, σ_0 = 169.08 ppm and m = 0.970; for ^{15}N, σ_0 = –159.84 ppm and m = 1.006. In each case, the dashed line corresponds to δ_{iso}(expt) = δ_{iso}(calc).

Details of Results from Structure-Solution Calculations of Polymorph I of dG(C_{10})$_2$ from Powder XRD Data

As discussed in the paper, structure solution of polymorph I of dG(C_{10})$_2$ involved 32 independent calculations using the direct-space genetic algorithm (GA) technique implemented in the program EAGER. Each GA calculation used a population of 500 trial crystal structures, which was allowed to evolve for 500 generations. For each of the 32 independent GA calculations, the population obtained after the final generation was inspected to find the trial structure giving the best fit to the experimental powder XRD data, and this trial structure was considered as a potential structure solution. The quality of fit (between calculated and experimental powder XRD data) for the best-fit structure obtained in each of the 32 independent GA calculations is shown in Figure S4.

The two structures (ringed in green) giving the best quality of fit were found to represent essentially the same structure, particularly in terms of the relative orientations of neighbouring guanine rings, corresponding to the formation of an N–H···N hydrogen bond between N7 and N10. The structure giving the next best quality of fit (ringed in red) had a similar orientation of the two C_{10} chains to those in the two best-fit structures, but the orientation of the guanine ring was different (in particular, this structure did not have N–H···N hydrogen bonding between N7 and N10 in neighbouring molecules). In line with common practice, the best-fit structure was considered as the best candidate structure solution, with the additional validation that this structure exhibits N–H···N hydrogen bonding between N7 and N10, which has already been demonstrated from solid-state ^{15}N NMR to exist in polymorph I of $dG(C_{10})_2$ (see discussion in the main text). On this basis, the trial structure giving the best quality of fit (R_{EAGER} = 9.64% in Figure S4) from all the structure-solution calculations was used as the initial structural model for Rietveld refinement.

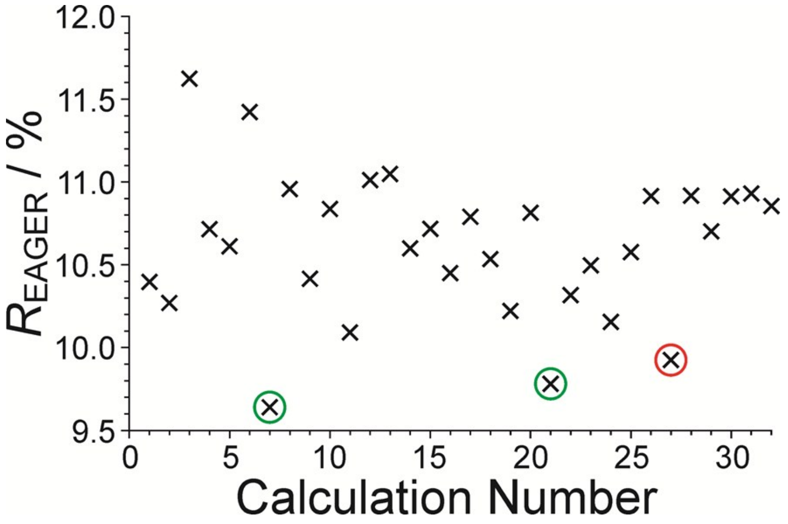

Figure S4. Value of the goodness-of-fit parameter R_{EAGER} for the best-fit structure obtained in each of the 32 independent GA structure-solution calculations for polymorph I of $dG(C_{10})_2$. The lowest value of R_{EAGER} obtained in these calculations was 9.64%. The definition of R_{EAGER} is very similar to the conventional weighted powder profile R-factor R_{wp}, but considers the experimental powder XRD data with baseline subtracted and has a slightly modified weighting scheme. If the Le Bail fitting and final Rietveld refinement calculations had used the experimental powder XRD data with baseline subtracted, the corresponding values of R_{EAGER} would have been 7.29% (Le Bail) and 8.42% (Rietveld).

Additional Tables

Table S1. Hydrogen bond geometries in the crystal structure of polymorph I of dG(C_{10})$_2$.

Hydrogen Bond	Length	Angle
N10–H···O	N···O, 3.11 Å	N–H–O, 143.3°
N1–H···O	N···O, 3.00 Å	N–H–O, 151.2°
N10–H···N7	N···N, 2.91 Å	N–H–O, 153.7°

Table S2. Calculated and experimental ^{1}H chemical shifts[a] for polymorph I of dG(C_{10})$_2$.

	$\delta(^1\mathrm{H})$ / ppm			$\delta(^1\mathrm{H})$ / ppm	
^{1}H Site	Calculated	Experimental[b]	^{1}H Site	Calculated	Experimental[b]
1	10.66	10.5			
8	8.48	8.7			
10a	9.77	8.5			
10b	7.57	6.8			
1'	7.27	6.6			
2'a	3.73	4.0			
2'b	1.90	2.2			
3'	6.52	6.0			
4'	3.65	3.6			
5'a	4.39	4.3 [c]			
5'b	3.40	4.3 [c]			
8'1a	0.97		12'1a	2.47	
8'1b	2.25		12'1b	1.29	
8'2a	0.58		12'2a	0.49	
8'2b	0.59		12'2b	–0.33	
8'3a	0.51		12'3a	–0.19	
8'3b	–0.44		12'3b	0.08	
8'4a	0.34		12'4a	0.41	
8'4b	0.23	0.8 [d]	12'4b	0.06	0.8 [d]
8'5a	0.25		12'5a	0.16	
8'5b	–0.17		12'5b	0.40	
8'6a	0.54		12'6a	0.08	
8'6b	–0.28		12'6b	0.13	
8'7a	0.73		12'7a	0.03	
8'7b	–0.27		12'7b	0.21	
8'8a	0.12		12'8a	0.05	
8'8b	0.86		12'8b	0.09	
9'a	0.20		13'a	0.25	
9'b	–0.50	0.5 [e]	13'b	–0.01	0.5 [e]
9'c	0.07		13'c	–0.18	

[a] Reference shift was 30.04 ppm.
[b] Taken from Webber *et al.* (2011).[29]
[c] Only one ^{1}H resonance is observed for 5'.
[d] Average for alkyl CH_2 resonances.
[e] Average for CH_3 resonances.

Table S3. Calculated and experimental ^{13}C chemical shifts[a] for polymorph I of $dG(C_{10})_2$.

	δ(^{13}C) / ppm	
^{13}C Site	Calculated	Experimental[b]
2	150.80	153.6
4	152.45	152.4
5	120.31	117.8
6	155.90	158.5
8	140.96	140.4
1'	90.99	86.7
2'	36.24	39.0
3'	78.14	77.0
4'	86.83	84.9
5'	62.26	64.9
7'	177.04	172.6
8'1	33.25	
8'2	22.20	
8'3	32.43	
8'4	32.28	23.2 – 34.6[c]
8'5	31.30	
8'6	28.13	
8'7	30.33	
8'8	21.24	
9'	9.87	15.0
11'	178.15	174.3
12'1	33.42	
12'2	24.38	
12'3	29.49	
12'4	31.08	23.2 – 34.6[c]
12'5	31.45	
12'6	30.78	
12'7	31.85	
12'8	23.55	
13'	11.77	14.6

[a] Reference shift was 171.09 ppm.
[b] Taken from Webber *et al.* (2011).[29]
[c] Range of alkyl CH_2 resonances.

Table S4. Calculated and experimental ^{15}N chemical shifts[a] for polymorph I of $dG(C_{10})_2$.

	δ(^{15}N) / ppm	
^{15}N Site	Calculated	Experimental[b]
1	–236.92	–235.4
3	–209.75	–210.3
7	–146.87	–144.4
9	–197.91	–201.3
10	–301.76	–301.8

[a] Reference shift was –160.19 ppm.
[b] Taken from Pham *et al.* (2007).[30]
